# Determination of Chlorinity of Water without the Use of Chromate Indicator

**DOI:** 10.1155/2010/602939

**Published:** 2011-03-02

**Authors:** Tae-Kee Hong, Myung-Hoon Kim, Myung-Zoon Czae

**Affiliations:** ^1^Department of Chemistry, Hanseo University, Seosan, Choongnam 139-743, Republic of Korea; ^2^Department of Sciences, Georgia Perimeter College, Dunwoody, GA 30338, USA; ^3^Department of Chemistry, Hanyang University, Seoul 133-791, Republic of Korea

## Abstract

A new method for determining chlorinity of water was developed in order to improve the old method by alleviating the environmental problems associated with the toxic chromate. The method utilizes a mediator, a weak acid that can form an insoluble salt with the titrant. The mediator triggers a sudden change in pH at an equivalence point in a titration. Thus, the equivalence point can be determined either potentiometrically (using a pH meter) or simply with an acid-base indicator. Three nontoxic mediators (phosphate, EDTA, and sulfite) were tested, and optimal conditions for the sharpest pH changes were sought. A combination of phosphate (a mediator) and phenolphthalein (an indicator) was found to be the most successful. The choices of the initial pH and the concentration of the mediator are critical in this approach. The optimum concentration of the mediator is *ca.* 1~2 mM, and the optimum value of the initial pH is *ca.* 9 for phosphate/phenolphthalein system. The method was applied to a sample of sea water, and the results are compared with those from the conventional Mohr-Knudsen method. The new method yielded chlorinity of a sample of sea water of (17.58 ± 0.22) g/kg, which is about 2.5% higher than the value (17.12 ± 0.22) g/kg from the old method.

## 1. Introduction

Chlorinity is one of the most fundamental quantities associated with water quality [1, 2], and it is directly related to the salinity of sea water [3] and often used to determine the salinity [4]. In recent years, physical methods of determining salinity [5, 6], such as coulometry, measurements of conductivity, refractive index, or density, have become more popular because of their speed. Nevertheless, chemical measurements of chlorinity still remains an important and independent method of characterizing water quality. Various chemical methods have been reported for chlorinity determination: the gravimetric method with AgCl precipitates [7–9], various volumetric precipitation titrations with silver (I) or mercury (II) ions [7–9], and a recent spectroscopic method utilizing the Raman scattering band of OH stretching of water [10]. Among these chemical methods, the volumetric titrations with precipitation with Ag^+^ have been most popular because it is less time consuming than the gravimetry. Several different ways of determining the equivalence point have been reported for the volumetric titrations with silver ion, such as (a) use of various visual indicators [11, 12], (b) thermometric titration measuring enthalpy changes [13], and (c) potentiometric titration [14]. The analytical methods recommended in the *Standard Method for the Examination of Water and Waste Water* [15] and the *Official and Standardized Method of Analysis* [16] are: (1) argentometric titration with silver nitrate using potassium chromate indicator, (2) titration with mercuric nitrate using s-diphenylcarbazone indicator, and (3) potentiometric titration using a glass and a silver-silver chloride electrode. The present work is aimed at improving the common volumetric method (1) of argentometric titration. However, the argentometry with the Mohr or Mohr-Knudsen method [1, 5, 7], which is based on appearance of the red color of silver chromate precipitate at the equivalence point raises concerns with environment problems because of the toxicity of the chromate with a hexavalent chromium.

 Although trivalent chromium, Cr(III), is nontoxic, the acute and chronic toxicity and carcinogenicity of the hexavalent chromium, Cr(VI), have been well documented [17–19]. Thus, conversion of Cr(VI) to Cr(III) and speciation and fate of chromium in the environment and in model systems and kinetics of reduction of Cr(VI) have drawn much interest in recent years [20–25]. Chromium pollution in surface water is largely due to discharge from chemical plants where chromium is used in tanning leather, as a mordant in the textile industry, and in the galvanic process for anodizing aluminum in the aircraft industry and other industries. The current Maximum Contaminant Level (MCL) of chromium from the EPA (USA) is 100 ppb (for water), and the Permissible Exposure Limit (PEL) from the OSHA (USA) is 52 *μ*g/m^3^ (for air). The latter is much higher than a new proposed level of 1 *μ*g/m^3^ [26]. Thus, it is desirable to reduce chromium discharge to environment in all possible ways. The toxicity of mercury is also well known [18]: thus, the method (2) is not a desirable one. With this in mind, we developed an environmentally more benign method for determining the chlorinity of water without using the hexavalent chromium or divalent mercury salt. 

 Changes in pH during certain types of precipitation titrations have long been observed [12, 27, 28], and the pH change can be made large enough so that an equivalence point can be determined either potentiometrically with a pH electrode or using acid-base indicators under suitable conditions. When reactions involve hydrolysis of a cation or an anion yielding a large pH, an equivalence point can be detected potentiometrically [29–31]. A quantitative model to predict the pH change in such hydrolytic systems have been reported [32, 33]: Dobcnik and coworkers proposed a mathematical model for the titration of a metal ion (Pb^2+^) with oxalate and other anions [34]. In their studies, after all of the lead is removed as lead oxalate precipitates at the equivalence point, the excess oxalate anions pick up H^+^ from solution to form oxalic acid: this triggers a rapid increase in pH. The equivalence point was determined to be a crossing point (i.e., the point at which two tangents on the two earlier sections of the titration curve intersect) [32–34]. Thus, the theory and practice of hydrolytic types of precipitation reaction have been established. A theory and practice for the precipitation titration *that does not involve a hydrolysis*, however, have not been well studied yet except in our recent report [35]. The argentometric titration of chloride *per se* does not involve hydrolysis of an anion or a cation because neither Ag^+^ nor Cl^−^ hydrolyses. Thus, very little change in pH (ΔpH < 0.1) has been observed around an equivalence point during a titration of chloride ion with silver ion [27]. In the presence of various adsorption indicators, however, somewhat larger changes in pH have been observed [12, 28]; typically, ΔpH is less than three units. This change is not large enough for an acid-base indicator to respond sharply at the equivalence point although it can be followed potentiometrically with a pH meter [36]. In our recent work [35], we have fully demonstrate, both in theory and practice, that the pH change at the equivalence point can be made sufficiently large even though the precipitation reaction does not involve hydrolysis. This was possible by introducing an additional reagent (chromate, a mediator) that undergoes hydrolysis so that the concentration of H^+^ can be changed drastically at the equivalence point. Equivalence points were detected *with a pH meter,* which yielded less than 1% of relative errors that depend on the mediators [35]. For clarity, a brief comparison with the old Mohr method and current new method is presented below in terms of the reaction involved.

### 1.1. Comparison of the Current Approach with the Mohr Method

The Mohr method utilizes the formation of a red-colored precipitate of titrant (Ag^+^) with an indicator (CrO_4_^−2^) (see (2)) after all the chloride is precipitated out of the solution by Ag^+^ (see (1)) at the equivalence point

(1) $$\begin{array}{c} {\text{Cl}}^{-}+{\text{Ag}}^{+}⇆\text{AgCl}\left(\text{s}\right)\;\left(\text{white}\right)\;\text{Analyte}\;\text{reaction} \end{array}$$

(2) $$\begin{array}{c} {{\text{CrO}}_{4}}^{-2}\left(\text{yellow}\right)+2{\text{Ag}}^{+}⇆{\text{Ag}}_{2}{\text{CrO}}^{4}\left(\text{s}\right)\;\left(\text{red}\right)\\ \;\;\;\;\;\;\;\;\;\;\text{Indicator}\;\text{reaction} \end{array}$$

The appearance of the red precipitate of silver chromate signals the end point of the titration. This method has been particularly useful in the determination of chlorinity [1, 4, 37] in samples of sea water. 

 In the current method [35], *a weak acid (HA) whose conjugate base (A* ^−^ *) can form a slightly soluble salt (AgA) with the titrant (Ag^+^) is added in place of the visual indicator (chromate) above*; this allows equivalence point to be detected either with a pH electrode [35] or with an acid-base indicator. The acid (HA) or its conjugate base (A^−^) acts as a mediator (or as an indicator) so that HA can release H^+^ when the equivalent point is reached. This system presents an equilibrium problem in which an acid-base reaction is coupled to two solubility equilibria, resulting in four coupled equilibria including the ionization of water (see (6))

(3) $$\begin{array}{c} {\text{Cl}}^{-}+{\text{Ag}}^{+}⇆\text{AgCl}\left(\text{s}\right)\;\left(\text{white}\right)\;\;{\text{K}}_{\text{sp}}\;=\;\left[{\text{Ag}}^{+}\right]\left[{\text{Cl}}^{-}\right], \end{array}$$

(4) $$\begin{array}{c} \text{HA}⇆{\text{H}}^{+}+{\text{A}}^{-}\;\;{\text{K}}_{\text{a}}=\frac{\left[{\text{H}}^{+}\right]\left[{\text{A}}^{-}\right]}{\left[\text{HA}\right]}, \end{array}$$

(5) $$\begin{array}{c} {\text{A}}^{-}+{\text{Ag}}^{+}⇆\text{AgA}\left(\text{s}\right)\;\;{\text{K}}_{\text{sp}}^{\prime }=\left[{\text{Ag}}^{+}\right]\left[{\text{A}}^{-}\right], \end{array}$$

(6) $$\begin{array}{c} {\text{H}}_{2}\text{O}⇆{\text{H}}^{+}+{\text{OH}}^{-}\;\;{\text{K}}_{\text{w}}=\left[{\text{H}}^{+}\right]\left[{\text{OH}}^{-}\right] \end{array}.$$

If the solubility of AgCl is less than that of AgA, AgCl will be precipitated out first. After all the Cl^−^ is removed, then additional Ag^+^ will react with A^−^ to remove it as a precipitate (AgA). As A^−^ is being removed, HA must dissociate to replenish A^−^, thereby generating H^+^. *Therefore, the pH of the system decreases at the equivalence point *[35].

In this present work, we searched for the best nontoxic mediator and the best conditions that can bring a larger and sharper pH change so that even a common acid-base indicator can be employed in detecting an equivalence point for the titration. It is demonstrated that equivalence point is determined by using *a nontoxic mediator (phosphate)* and an acid-base indicator(phenolphthalein) *without using a pH meter*. This new approach is successfully applied for a determination of chlorinity of a sample of sea water.

## 2. Experimental

### 2.1. Reagent and Apparatus

All chemicals used were analytical reagent grade and were used without further purification. All solutions were prepared with deionized water. AgNO_3_ solutions were standardized using the Mohr method [7–9]. pH values were measured with a Fisher Accumet Selective Ion Analyzer Model 750 pH Meter and an Orion Model 810 Digital pH Meter. Glass electrodes of Orion Model-91 series and a similar type of combination electrodes were used for the pH measurements. Initial pHs were adjusted by adding 0.10 M NaOH or 0.1 M HCl. Solutions were stirred magnetically during titration.

## 3. Results and Discussion

**Figure 1:**
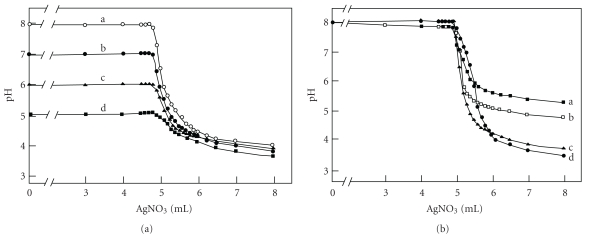
(a) Titration curves for 25.0 mL of 0.10 M NaCl with 0.50 M AgNO_3_ in the presence of 1.3 mM phosphate at various starting pH values: (a) 7.9, (b) 7.0, (c) 6.0, and (d) 5.1. (b) Titration curves for 25.0 mL of 0.10 M NaCl with 0.50 M AgNO_3_ in the presence of various phosphate concentrations: (a) 0.05, (b) 0.20, (c) 2.0, and (d) 6.0 mM.

Figure 1(a) presents titration curves for 25.0 mL of 0.10 M NaCl with 0.50 M AgNO_3_ in the presence of a mediator (1.3 mM phosphate) at various pH values of 7.9, 7.0, 6.0, and 5.1. As predicted from the theory, the curves have same pattern as Figure  3(a) in [35]. At the highest initial pH values, the change in pH is the most with 3.9 pH units, and at the lowest initial pH, the change is the smallest with 1.4 pH units. The pH changes are summarized in Table 1. The crossing points for all the curves occurred somewhat earlier than the equivalence point.

Figure 1(b) presents titration curves for the same solutions at various phosphate concentrations (0.05, 0.20, 2.0, and 6.0 mM). Again, the pattern resembles those predicted from the theory [35]. The crossing points for all the curves occur very close to the equivalence points. At lower mediator concentration, the change in pH is less (ΔpH = 2.5 with curve (a)), and at higher mediator concentration the change is the largest (ΔpH = 4.4 with curve (d)). Table 2 summarize the pH change at various concentration of the mediator.

**Figure 2:**
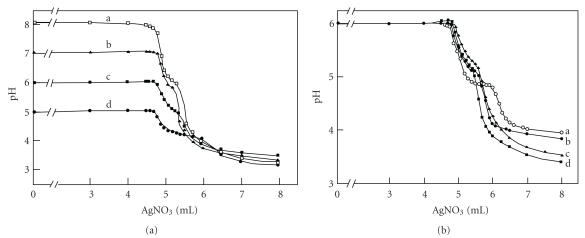
(a) Potentiometric titration curves for 25.0 mL of 0.10 M NaCl with 0.50 M AgNO_3_ in the presence of 3.2 mM EDTA at various starting pH values: (a) 8.1, (b) 7.0, (c) 6.0, and (d) 5.0. (b) Titration curves for 25.0 mL of 0.10 M NaCl with 0.50 M AgNO_3_ at various concentrations of EDTA: (a) 0.70, (b) 3.2, (c) 6.3, and (d) 14.2 mM.

Figure 2(a) presents titration curves for the same solutions with another mediator, 3.2 mM EDTA, at various starting pH values: (a) 8.1, (b) 7.0, (c) 6.0, and (d) 5.0. Although it exhibits the general trend, there are breaks in the curves. At the higher pH (8.1 and 7.0), HY^3−^ is dominant, and at the lower pH (5.0 and 6.0), H_2_Y^2−^ is dominant at the beginning. The one begun at 8.1 yielded the largest pH change. The narrow middle plateau for the curves (a)–(d), where the pH change becomes gradual again, is attributed to the conversion of either HY^3−^ to H_2_Y^2−^ or H_2_Y^2−^ to H_3_Y^−^. The crossing point occurred at about 4.7 mL, which is about 6% error from the equivalence point. Thus, in the case of EDTA; however, the inflection points at about 4.9 mL appear to be a better equivalence point (with 2% of error) than the crossing point. 

Figure 2(b) presents titration curves for the same solutions at various EDTA concentrations ranging from 0.7 (a) –6.3 mM (d). The one with the highest concentration (6.3 mM) yielded the largest pH change. The crossing point for all curves has an error of 6%. Thus, as in Figure 2(b), the inflection point appears to be a better equivalence point than the crossing points. Thus, EDTA does appear to be a good mediator in determining an equivalent point with the crossing point method. 

**Figure 3:**
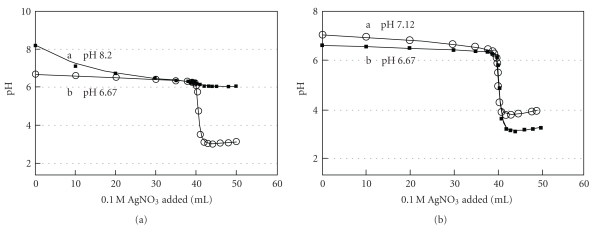
(a) Titration curves of 40.00 mL of 0.1 M NaCl with a 0.1 M AgNO_3_ solution at two different bisulfite concentrations: (a) 0.50 mM (■, initial pH 8.20), (b) 5.0 mM (○, initial pH 6.67). (b) Titration curves of 40.00 mL of 0.1 M NaCl with a 0.1 M AgNO_3_ solution in the presence of 5.0 mM bisulfite at two different initial pH values: (a) 7.12 (○), (b) 6.67 (■).

**Figure 4:**
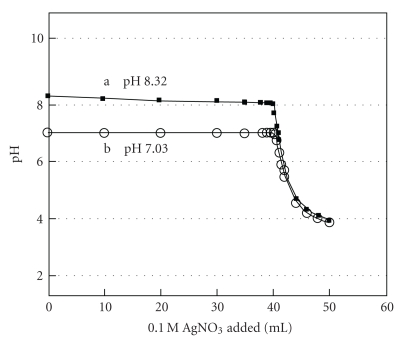
Titration curves of 40.0 mL of 0.1 M NaCl with a 0.1 M AgNO_3_ solution in the presence of 2.5 mM Na_2_HPO_4_ at two different initial pH values: (a) 8.32 (■) and (b) 7.03 (○).

All the previous experiments were performed as rather crude qualitative pilot runs using 0.50 M AgNO_3_. However, the following titrations (Figures 3 and 4) are aimed at obtaining more accurate quantitative results by using 0.10 M AgNO_3_ instead of 0.50 M AgNO_3_, and by increasing volumes of titrand (Cl^−^) from 25.0 mL to 40.0 mL. Typical titration curves of 40.0 mL of 0.1 M NaCl with a 0.1 M AgNO_3_ solution in the presence of NaHSO_3_ (the mediator) are presented in Figure 3(a) at two different concentrations of bisulfite: (a) 0.50 mM (■, initial pH of 8.2) and (b) 5.0 mM (○, initial pH of 6.67). 

The two results from the two concentrations are very different. The one obtained at 5.0 mM bisulfite generated a curve with a well-defined equivalence point. Meanwhile, the one with a bisulfite concentration of 0.5 mM generated a gradual pH change from the beginning without any break in pH changes, thus failing to produce a measurable crossing point. This illustrates the importance of controlling the concentration of the mediator and initial pH values. At the lower mediator concentration (0.5 mM), most of the bisulfite exists in the fully deprotonated form (SO_3_^2−^) at the pH of 8.2. Therefore, consumption of the sulfite by Ag^+^ cannot drive a reaction (HSO_3_^−^→ H^+^ + SO_3_^2−^) to release much H^+^. Similar results of gradual pH change at the equivalence point, without any sharp break, were observed when NaCN was used as a mediator.

Figure 3(b) shows titrations in the presence of 5.0 mM bisulfite at two different initial pH values ((a) 7.12, and (b) 6.67). Both yielded comparably good crossing points. A suitable range of initial pH values lies around 6 and 7. When several mM of NaHSO_3_ are added, as a mediator, the initial pH of the solution will be in the right range, thus adjustment of pH at the beginning is not necessary. However, when H_2_SO_3_ is added as a mediator, it will be acidic (pH *∼* 2) at the beginning; hence, a small amount of base (0.1 M NaOH) must be added to reach the optimum pH range. If Na_2_SO_3_ is used, the solution will be alkaline (pH *∼* 9); therefore, an acid solution (0.1 M HNO_3_) should be added to bring pH value of the alkaline solution down to a range of 6*∼*7. Optimum concentration of the mediator was found to be in the range of 2*∼*5 mM: a lower concentration of the mediator generates a small and gradual change in pH, while a higher concentration will generate less sharp changes in pH at the end point yielding a larger error. It is not clear why the pH increases again after equivalence point (after 41 mL). 

Figure 4 shows titration curves of 40.0 mL of 0.1 M NaCl with a 0.1 M AgNO_3_ solution in the presence of 2.5 mM Na_2_HPO_4_ at two different initial pH values: (a) 8.32 (■) and (b) 7.03 (○). Even though the titration with the higher initial pH yielded a larger and sharper pH change, both results were comparably good. Table 3 presents results with phosphate at several different conditions. The crossing point method yielded much better results (an average of 0.1% relative error) than the inflection methods.

Finally, we applied the present method with an acid-base indicator to determine the chlorinity of a sample of sea water, and we compared the results of this method with the results from the Mohr-Knudsen Method. The phosphate was adopted as a mediator; phenolphthalein was employed as a visual indicator. The results are summarized in Table 4. Five trials were made; the average and standard deviation values were (17.12_4_ ± 0.22_4_) g/kg from the Mohr-Knudsen method and (17.58_4_ ± 0.21_9_) g/kg from the present method. The average chlorinity from the mediator method was about 2.5% higher than that from the conventional method; the precision is about the same for both methods. The *F*-test and *t*-test yielded *F* = 0.9559 (<6.39), *t* = 3.31, respectively, suggesting that the two standard deviations do not differ, but the results (chlorinities) are different somewhat (2.5%). The higher value observed with the new method may be explained from the fact that the new method involves equilibrium (2), instead of equilibrium (1) in the old method: the additional equilibrium reaction may have required more Ag^+^ for a complete shift of the equilibrium reaction to the right. Both values of the chlorinity of the sample (collected at the Western Sea of South Korea) are about 1% lower than that of an average chlorinity of sea water which is 19 g/kg [38, 39] that depends on a location of sampling.

## 4. Summary

A new method for determining chlorinity of water without using the chromate indicator was developed and successfully applied to determine chlorinity of sea water. Among the mediators tested, the phosphate in combination with phenolphthalein yielded the best result. Concentration of a mediator and the initial pH of the solution found to be critical for the success. Higher mediator concentrations yielded less accurate results even though they gave larger changes in pH. At lower mediator concentration, the end point is more accurate, but pH change is not large enough for the visual indicator to bring a sharp color change. Thus, optimum concentration of the phosphate mediator appears to be in a range of 1*∼*2 mM. The pH of the analyte solution must be adjusted initially to the alkaline side (pH 8.5 or higher) so that phenolphthalein imparts a pink color at the beginning of the titration. Chlorinity of a sample of sea water from this method found to be (17.58 ± 0.22) g/kg, which is about 2.5% higher than that (17.12 ± 0.22) g/kg from the conventional Mohr-Knudsen method. This new method may replace the argentometric titration with chromate indicator in the standard method [15] of determining chloride in water and waste water.

## Figures and Tables

**Table 1:** Change in pH at various values of initial pH with Na_2_HPO_4_.

Initial pH	pH range	ΔpH
5.1	5.1–3.7	1.4
6.0	6.0 –3.8	2.2
7.0	7.0 –3.9	3.1
7.9	7.9–4.0	3.9

**Table 2:** Change in pH at various concentrations of Na_2_HPO_4_.

Concentartion (mM)	pH range	ΔpH
0.05	7.8–5.4	2.4
0.20	7.8–4.8	3.0
2.0	8.0–3.8	4.2
5.0	8.0–3.6	4.4

**Table 3:** Errors in determining equivalence point: volume (mL) of Ag^+^ required using Na_2_HPO_4_.

Trial	Mediator (mM)	Initial. pH	Crossing Point	Inflection Point
1	1.25	8.10	40.0	41.5
2	1.25	8.10	39.9	41.5
3	2.50	8.32	40.0	41.1
4	2.50	7.20	39.9	41.1
5	2.50	7.10	40.0	41.7
6	2.50	7.00	40.0	41.7

Average of Rel. Error			0.1 %	3%

**Table 4:** Comparison of the Results of Determination of Chlorinity (g/kg) from the Mohr-Knudsen Method and the Present Methods with Phosphate/Phenolphthalein as Indicator.

Trial	Mohr-Knudsen Method	Phosphate/Ph'pht Method (Present)	Difference
1	17.06	17.42	0.38
2	17.00	17.32	0.32
3	16.85	17.87	1.02
4	17.36	17.61	0.05
5	17.35	17.70	0.35

Ave. ± Std.Dev.	17.12 ± 0.22	17.58 ± 0.22	
